# Isolation Cocoon, May 2020—After Zhuangzi’s *Butterfly Dream*

**DOI:** 10.3201/eid2611.202993

**Published:** 2020-11

**Authors:** Ron Louie

**Affiliations:** University of Washington, Seattle, Washington, USA; Mary Bridge Children's Hospital and Health Center, Tacoma, Washington, USA

**Keywords:** COVID-19, 2019 novel coronavirus disease, SARS-CoV-2, severe acute respiratory syndrome coronavirus 2, viruses, respiratory infections, zoonoses, coronavirus, isolation, quarantine

Spinning, what you will, in heeding that swarm of guidance, creating

your own shell, then transforming, as you will, within that isolation,

still seems like an almost unconvincing, almost unnecessary nuisance.

You had chosen this situation, if it is fair to say there was a choice,

when there was no viable alternative. Your cocoon can feel so safe,

an illusion perhaps, but reality provides nothing less vulnerable.

The walls are thin enough to allow you to breathe, and to vaguely hear

or feel vibrations, even though their meaning cannot be known.

Light penetrates, and darkness, too; the changes remain obscure.

Ruminating on that former lifestyle, you can digest time thoroughly,

like those last memorable green leaves of Springtime, then so succulent,

and satisfying, but to what end you know not; not all cocoons survive.

Time, space, being, identity, the interpreted past, the fancied future

can all be consumed within your insatiable capsule. Chrysalis or cocoon,

distinctions no longer matter; each benefits from a covering and distancing.

Complacency or contentment allows a concentration on one’s only

certainty, the presentness right now, in this cell-like confinement,

because emergence would require several just preposterous miracles.

